# *Erk1* Positively Regulates Osteoclast Differentiation and Bone Resorptive Activity

**DOI:** 10.1371/journal.pone.0024780

**Published:** 2011-09-22

**Authors:** Yongzheng He, Karl Staser, Steven D. Rhodes, Yaling Liu, Xiaohua Wu, Su-Jung Park, Jin Yuan, Xianlin Yang, Xiaohong Li, Li Jiang, Shi Chen, Feng-Chun Yang

**Affiliations:** 1 Departments of Pediatrics, Indiana University School of Medicine, Indianapolis, Indiana, United States of America; 2 Herman B Wells Center for Pediatric Research, Indiana University School of Medicine, Indianapolis, Indiana, United States of America; 3 Anatomy and Cell Biology, Indiana University School of Medicine, Indianapolis, Indiana, United States of America; Oklahoma Medical Research Foundation, United States of America

## Abstract

The extracellular signal-regulated kinases (ERK1 and 2) are widely-expressed and they modulate proliferation, survival, differentiation, and protein synthesis in multiple cell lineages. Altered ERK1/2 signaling is found in several genetic diseases with skeletal phenotypes, including Noonan syndrome, Neurofibromatosis type 1, and Cardio-facio-cutaneous syndrome, suggesting that MEK-ERK signals regulate human skeletal development. Here, we examine the consequence of *Erk1* and *Erk2* disruption in multiple functions of osteoclasts, specialized macrophage/monocyte lineage-derived cells that resorb bone. We demonstrate that Erk1 positively regulates osteoclast development and bone resorptive activity, as genetic disruption of *Erk1* reduced osteoclast progenitor cell numbers, compromised pit formation, and diminished M-CSF-mediated adhesion and migration. Moreover, WT mice reconstituted long-term with *Erk1^−/−^* bone marrow mononuclear cells (BMMNCs) demonstrated increased bone mineral density as compared to recipients transplanted with WT and *Erk2^−/−^* BMMNCs, implicating marrow autonomous, Erk1-dependent osteoclast function. These data demonstrate Erk1 plays an important role in osteoclast functions while providing rationale for the development of Erk1-specific inhibitors for experimental investigation and/or therapeutic modulation of aberrant osteoclast function.

## Introduction

Normal bone physiology, as well as bone repair following injury, depends upon the productive and destructive interactions between osteoblasts and osteoclasts, whereby osteoblast-mediated bone production repairs localized defects created by osteoclasts. Many skeletal diseases result from an imbalance between osteoclast and osteoblast numbers and/or function [1]. Generally, increased osteoblast numbers and/or functions lead to abnormal bone mineralization while increased osteoclast numbers and/or functions underlie osteoporotic conditions. Multinucleated osteoclasts are continuously formed from the monocyte/macrophage lineage of hematopoietic cells [2], [3], and osteoclastogenesis and normal osteoclast function depend upon multiple cytokines and growth factors, including macrophage colony stimulating factor (M-CSF), receptor activator for nuclear factor kappa-B ligand (RANKL), and transforming growth factor beta (TGFβ) [4], [5], [6]. These factors near-ubiquitously induce phosphorylation of Erk1 and Erk2 [4], [5], [6]. Intriguingly, alterations in the MAPK/ERK1/2 cascade contribute to cardio-facio-cutaneous syndrome (CFC), Noonan syndrome, LEOPARD syndrome, and Neurofibromatosis type 1. These pathophysiological observations suggest roles for ERK1 and ERK2 in human skeletal development [7], [8], [9].

ERK1 and ERK2 are serine/threonine-specific protein kinases sharing 84% homology [10], [11]. Upon extracellular mitogen stimulation, the Ras-Raf-MEK cascade phosphorylates and activates ERK1 and ERK2, which then phosphorylate cytoplasmic and nuclear factors that execute normal and malignant cell functions, including gene expression, mitosis, movement, and the regulation of apoptosis [12], [13], [14], [15], [16]. *In vivo* genetic studies have shown differential and/or combined roles for Erk1 and Erk2 in multiple cell lineages, including T cells, B cells, and osteoblasts [17], [18], [19], [20], [21], [22], [23]. Specifically, dual *Erk1/2* disruption in osteoblast lineages results in reduced RANKL production, leading to a subsequent reduction in osteoclastogenesis. Likewise, studies using pharmacologic kinase inhibitors (e.g. U0126, Wortmannin, LY294002) have suggested the importance of these pathways to osteoclast formation and function [24], [25]. However, no genetic study has examined Erk1 or Erk2's direct regulation of osteoclast physiology, although recent inquiries in other cell lineages have discovered isoform-specific roles for Erk1 and Erk2 [7], [17], [23].

In the present study, we evaluated the consequence of *Erk1* and *Erk2* disruption in osteoclast differentiation, migration, and bone resorptive activity *in vitro* and *in vivo*. To accomplish these aims, we performed functional assays on osteoclasts generated from bone marrow progenitor cells of *Erk1^−/−^* and hematopoietic *Erk2* conditional knockout (*Mx1Cre* ^+^ *Erk2^flox/flox^*) mice, and we assessed bone mineral density in WT mice transplanted long-term with bone marrow mononuclear cells (BMMNCs) of WT, *Erk1^−/−^*, or *Erk2^−/−^* mice. We conclude that Erk1 plays a preponderant role in modulating osteoclast differentiation, migration, bone resorption, and bone mineral density.

## Materials and Methods

### Animals

Previously described *Erk1^−/−^* mice and *Erk2^flox/flox^* mice [18], [22] were crossed with *Mx1Cre* transgenic mice (*Mx1Cre^+^ Erk2^fl/fl^*), allowing inducible disruption of *Erk2* in all hematopoietic cells. Cre expression was induced by intraperitoneal injections of poly I poly C (polyIC) (300 µg at 1 mg/mL in PBS; Sigma) every two days for a total of five injections. Genetic disruption of the *Erk2* gene in *Mx1Cre^+^* mice was verified by PCR of the recombinant *Erk2* gene and the absence of total Erk2 protein by western blot of mouse BMMNCs (Figure S1 and Figure S2, respectively). For simplicity, mice containing the disrupted *Erk2^flox/flox^* allele henceforth will be referred to as *Erk2^−/−^* mice. Animal care and experiments were conducted according to the guidelines established by the Indiana University Animal Care and Use Committee (IACUC). Age- and sex-matched WT, *Erk1^−/−^*, and *Erk2^−/−^* mice were used for each experiment.

### Bone marrow transplantation

2×10^6^ BMMNCs from WT, *Erk1^−/−^* and *Erk2^−/−^* mice were injected intravenously into lethally-irradiated (1100 cGy) 8-week old BoyJ mice [26]. Successful marrow reconstitution was confirmed by flow cytometry of CD45.2 expression in peripheral white blood cells, and stable *Erk2* allele deletion was confirmed by western blot. Bone mineral density (BMD) was measured six months after bone marrow transplantation.

### BMD quantification

Bone mineral density (BMD) was measured by dual-energy X-ray absorptiometry (DEXA) with a Lunar Piximus densitometer (GE Medical Systems, software version 1.4 Lunar) [27]. The mice were anesthetized with avertin/tribromoethanol (0.25 mg/kg) and placed into the scanner in the prone position with arms and legs extended. The BMD of the left femoral metaphysis was measured by defining a region of interest of 11 pixels×10 pixels proximal to the distal growth plate, a region containing high content of trabecular bone.

### Micro computed tomography (μCT)

To evaluate trabecular microarchitecture in the distal femoral metaphysis, fixed femurs (stored in 70% EtOH) were scanned using a high-resolution desktop microcomputed tomography imaging system (μCT-20; Scanco Medical AG, Basserdorf, Switzerland). Scanning for the femur was started at 15% of the total femur length measured from the tip of femoral condyle and extended proximally for 200 slices with an increment of 9 µm, which were then reconstructed, filtered (σ = 0.8 and support = 1.0), and thresholded (at 22% of the possible gray scale value) for analysis, as described previously [28]. The trabecular region was outlined within the trabecular compartment, excluding the cortical shell. Parameters of microarchitecture for bone volume fraction (BV/TV, %), trabecular number (Tb.N, mm^−1^), trabecular thickness (Tb.Th, mm), as well as and trabecular separation (Tb.Sp, mm) were measured.

### Clonogenic progenitor assays

Colony-forming unit-macrophage/monocyte (CFU-M) of BMMNCs were assayed, as described previously [27]. Briefly, 2.5×10^4^ BMMNCs were seeded onto a 35-mm gridded dish containing methylcellulose supplemented with murine recombinant macrophage-colony stimulating factor (M-CSF, 30 ng/mL) and murine recombinant RANKL (20 ng/mL) for 7 days at 37°C in a 5% CO_2_ incubator. Colonies were scored using an inverted light microscope. All cytokines were purchased from PeproTech (Rocky Hills, NC).

### Generation of murine osteoclasts and tartrate resistant acid phosphatase (TRACP) staining

Mouse osteoclasts were obtained *in vitro* using BMMNCs, as described previously [27]. BMMNCs from 8-week old WT, *Erk1^−/−^*, and *Erk2^−/−^* mice were isolated by long bone marrow flush and Ficoll density gradient isolation and cultured in α-MEM supplemented with 10% fetal bovine serum (FBS, Sigma), 30 ng/mL M-CSF and 20 ng/mL RANKL for 3 days. On day 4, cell culture media was switched to α-MEM supplemented with 10% FBS, M-CSF (30 ng/mL), and 60 ng/mL RANKL for another 3 days. To identify osteoclasts after this culture period, adherent cells were fixed with a solution containing 25 mL citrate solution, 65 mL acetone, and 8 mL of 3.7% formaldehyde, and stained for TRACP. Osteoclasts were visualized with a Nikon TE2000-S microscope (Nikon Inc., Melville, NY). Images were taken by a QImaging camera and QCapture-Pro software (Fryer Company Inc., Cincinnati, OH). Multinucleated TRACP^+^ cells containing more than three nuclei were scored as mature osteoclasts. The area of multinucleated, TRACP^+^ osteoclasts and the number of nuclei per osteoclast were calculated using MetaMorph Offline software (Molecular Devices, Inc. Sunnyvale, CA).

### Bone resorption assay

Osteoclasts were dissociated from tissue culture plates using 0.5% Trypsin-EDTA. Single-cell suspensions of purified osteoclasts were seeded at a density of 1×10^4^/well on dentine slices (ALPCO Diagnostic, Windham, NH) pre-wetted with α-MEM for 2 hours and then incubated at 37°C/5% CO_2_ in the presence of 30 ng/mL M-CSF and 60 ng/mL RANKL, as previously described [27]. Following 7 days of culture, the slices were rinsed with PBS, immersed overnight in 1 M ammonium hydroxide, and stained with a 1% toluidine blue/0.5% sodium tetraborate solution. At the same time, cells on dentine slices in the independent cultures were fixed and stained with TRACP for osteoclast counting. Microphotographs were taken under a reflective light microscopy at a 100× magnification, and the resorptive areas or “pits” in low-power field were analyzed by the MetaMorph Offline software. Six fields per condition were scored.

### Migration assay

Migration of preosteoclasts was evaluated with a transwell assay, as described previously [27]. Equivalent numbers of cells were loaded onto the upper chamber of an 8 µm polycarbonate transwell (Corning Inc., Lowell, MA) coated with vitronectin (Takara, Japan) for 15 hours in a humidified incubator at 37°C/5% CO_2_. The lower chamber contained α-MEM supplemented with 0.1% bovine serum albumin and M-CSF (30 ng/mL). Cells that migrated to the bottom chamber were stained with crystal violet and the number of migrated cells per field was counted. Six fields per condition were counted.

### Preosteoclast adhesion assay

A single-cell suspension of osteoclast precursors (1×10^4^ cells/well) were placed into 96-well plates pre-coated with vitronectin as previously described [29]. After 30 minutes of incubation, nonattached cells were gently washed away with PBS and adherent cells were fixed and counted.

### Western blotting analysis

Western blot using phospho-specific antibodies was conducted to determine the phosphorylation levels of Erk1/2 and p90^rsk,^ (Cell Signaling, Danvers, MA) in preosteoclasts [29]. Phosphorylation levels were compared to total-ß-actin levels (Cell Signaling). Briefly, cells were deprived of growth factors for 12 hours in α-MEM supplemented with 0.5% BSA, stimulated with or without 30 ng/mL M-CSF for 5 minutes and lysed in ProteoJET lysis buffer (Fermentas, Glen Burnie, MD) supplemented with Complete Mini protease inhibitor cocktail (Roche, Indianapolis, IN). The intensity of the bands was measured with Fluorchem Software (Cell Biosciences, Inc., Santa Clara, CA) and standardized with beta-actin for arbitrary intensity comparison.

### Detection of C-terminal telopeptide of type I collagen (CTX) in plasma

Blood was collected from the retrobulbar venous plexus and kept in EDTA coated tubes, which were immediately cooled on ice and centrifuged within 30 min. Plasma samples were stored at −80°C before being tested. All samples were analyzed in the same experiment. CTX concentrations were determined using a commercial enzyme immunoassay kit (Ratlaps™ EIA, Immunodiagnostic systems Inc., Fountain Hills, AZ).

### Histomorphometric measurements

Upon sacrifice, femurs were harvested for histomorphometric analyses. The isolated bones were fixed in 10% neutral buffered formalin for 48 hours, dehydrated in graded ethanols, and embedded undecalcified in methyl methacrylate. Sagittal sections (5 µm thick) were cut from the middle of the femur. TRACP staining was performed using a leukocyte acid phosphatase kit (Sigma Diagnostics, St. Louis, Missouri) and McNeal Staining was performed using McNeal's Tetrachrome kit (Polysciences, Warrington, Pennsylvania), both according to manufacturers' protocols. One section per femur was viewed at 100× magnification on a Leitz DMRXE microscope (Leica Mikroskopie und System GmbH, Wetzlar, Germany) and the image captured using a QImaging camera and QCapture-Pro software (Fryer Company Inc., Cincinnati, OH). The measurement area for the metaphysis was determined by a region beginning 0.5 mm proximal to the midpoint of the growth plate, non-inclusive of cortical bone, and extending proximally for a total area of approximately 2.8 mm^2^.

### Alpha V (αv) integrin detection by flow cytometry

Expression of αv integrin was assessed by flow cytometry. Preosteoclasts were blocked using CD16/32 (BD Biosciences), washed and incubated with PE-linked anti-CD51 (αv integrin) antibody (BD Biosciences) for 60 min at 4°C. After three further washing steps, CD51 expression was measured using a FACSCalibur™ flow cytometer (Becton-Dickinson, San Jose, USA). Data were analyzed with FlowJo Software (version 7.6, TreeStar Inc.).

### Statistical analyses

Unpaired or paired student's *t*-tests or ANOVA with appropriate post hoc corrections were used, as indicated, to evaluate differences among genotypic groups. *P* values less than 0.05 were considered significant. Statistical analyses were performed with Prism 5.0 software (GraphPad, La Jolla, CA).

## Results

### Deletion of *Erk1* or *Erk2* in BMMNCs and cultured osteoclasts

PCR was conducted to assay the presence of the *Erk1* null allele (*Erk1^−/−^*), *Mx1Cre* transgene, and flanking loxP (*Erk2^flox/flox^*) alleles. Figure S1 depicts the genomic PCR analysis for homozygous null *Erk1* (600 bp) and homozygous floxed *Erk2* (432 bp).To induce Cre expression and genetic disruption of *Erk2*, mice were treated with polyIC, as described in the Materials and Methods. Western blot analysis demonstrated undetectable Erk1 or Erk2 in protein isolates from BMMNCs extracted from genomic-verified *Erk1* ^−/−^ or *Mx1Cre^+^ Erk2^flox/flox^* mutant mice (Figure S2, top panels). Likewise, western blot of protein from cultured osteoclasts demonstrated no detectable Erk1 or Erk2 protein (Figure S2 lower panels). For simplicity throughout, we refer to the genotypes of BMMNCs and osteoclasts derived from polyIC-treated *Mx1Cre^+^ Erk2^flox/flox^* mice as *Erk2^−/−^*.

### Decreased multinucleated osteoclast formation from *Erk1^−/−^* BMMNCs

**Figure 1:**
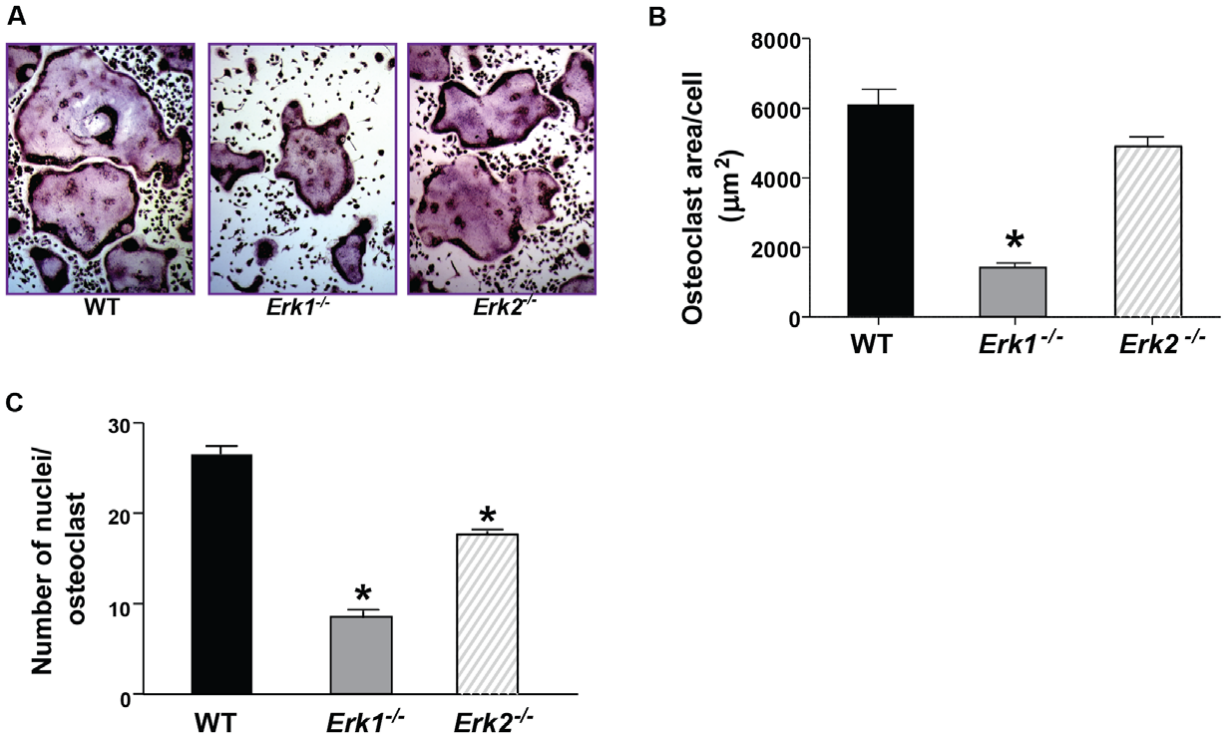
Genetic deletion of *Erk1* hinders osteoclast development. (**A**) Representative microphotograph of osteoclast formation of the indicated genotypes generated *in vitro* following culture in a-MEM, 10% FBS, M-CSF 30 ng/mL and RANKL 60 ng/mL for 6 days. Osteoclasts were identified by TRACP staining. (**B**) Quantitative analysis of the area of osteoclasts is shown. *P<0.01 for *Erk1^−/−^* vs. WT and *Erk2^−/−^* by ANOVA followed by post-hoc *t*-tests. (**C**) Quantitative analysis of the number of nuclei per osteoclast is shown. Data represents Mean ± SEM of six fields per condition in triplicates. Experiments were conducted on three independent occasions with similar results. *P<0.01 for *Erk1^−/−^* vs. WT and *Erk2^−/−^* vs. WT as assessed by ANOVA followed by post-hoc *t*-tests.

To evaluate the consequence of *Erk1* or *Erk2* disruption in osteoclast differentiation, we cultured osteoclasts from BMMNCs in M-CSF- and RANKL-supplemented α-MEM, followed by TRACP staining to evaluate cellular morphology. Compared with wildtype (WT) cultures, *Erk1^−/−^* BMMNCs demonstrated significantly decreased osteoclast-forming areas, as shown qualitatively and quantitatively in Figure 1A and 1B, respectively. In addition, the average number of nuclei per osteoclast was significantly less in *Erk1^−/−^* cultures than that in WT or *Erk2^−/−^* cultures (Figure 1C). Although we found no difference in the size of osteoclasts between *Erk2^−/−^* and WT cultures, the number of nuclei of *Erk2^−/−^* osteoclasts were slightly decreased as compared to WT osteoclasts. *Erk1^−/−^* cells display impaired osteoclast differentiation while Erk2 level should remain at least as WT cell level. It is possible that ERK2 expression in osteoclasts is not high enough to compensate for the loss of ERK1.

### Genetic deletion of *Erk1* impairs osteoclast migration and bone resorption

**Figure 2:**
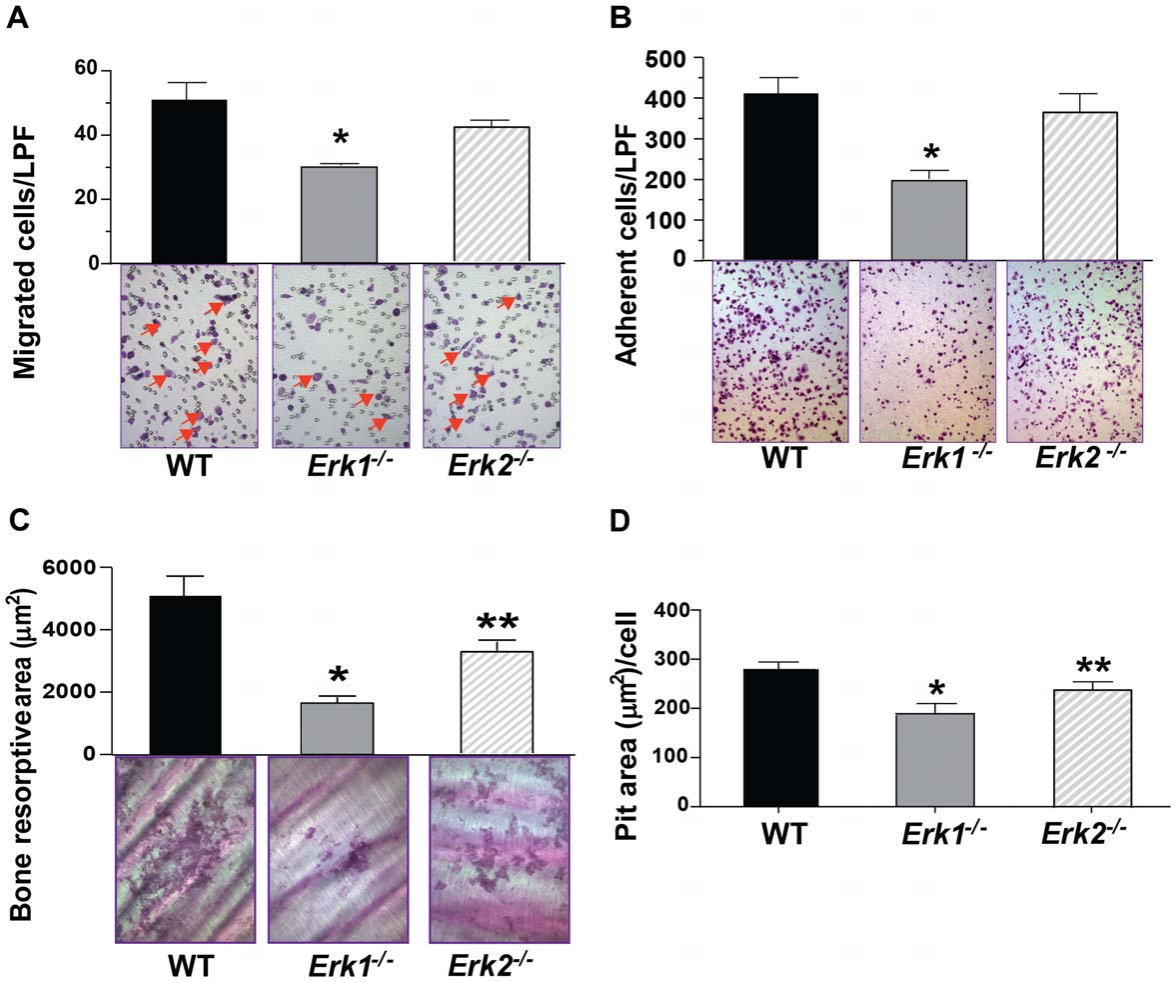
Effect of *Erk1* deletion on osteoclast haptotaxis, adhesion, and bone resorption in response to M-CSF. (**A**) Haptotaxis of preosteoclasts from WT, *Erk1^−/−^*, *Erk2^−/−^* cultures in response to M-CSF. Representative microphotographs of low power field (100× magnifications) from 1 of 3 experiments are shown. Quantitative evaluation of migration in response to M-CSF was performed. Result represents the mean ± SEM of six fields per condition in triplicates. Three independent experiments were conducted with similar results. *P<0.01 by ANOVA. (**B**) Quantitative evaluation of M-CSF mediated preosteoclast adhesion (30 minutes) to αVβ3 is shown. Data represents mean ± SEM of six fields per condition of triplicates. Representative microphotographs (100× magnification) are shown. *P<0.01 by ANOVA followed by post-hoc *t*-tests. Experiment conducted on three independent occasions with similar results. (**C**) Representative microphotographs of the bone resorption on dentine slices, referred to as ‘pits’, are shown. Bar graph indicates the average of “pit” area per dentine slice. Data represents mean+standard error of the mean (SEM). *P<0.01 for *Erk1^−/−^* vs. WT, **P<0.05 for *Erk2^−/−^* vs. WT and *Erk1^−/−^* as assessed by ANOVA followed by post-hoc *t*-tests. (**D**) Quantitative analysis of bone resorption following culture of osteoclasts on dentine slices. The area of the resorbed regions per osteoclasts was quantified. Data represents one of three independent experiments with similar results. *P<0.01 for *Erk1^−/−^* vs. WT, **P<0.05 for *Erk2^−/−^* vs. WT and *Erk1^−/−^* as assessed by ANOVA followed by post-hoc *t*-tests.

Osteoclast bone resorptive capacity requires that osteoclast progenitor cells (preosteoclasts) migrate across the bone surface. To evaluate whether genetic disruption of *Erk1* or *Erk2* affects preosteoclast migration *in vitro*, we assessed transwell migration of preosteoclasts in response to recombinant M-CSF, a chemotactic signal for monocytic cells [27], [29]. *Erk1^−/−^* preosteoclasts migrated at a lower number than both WT and *Erk2^−/−^* preosteoclasts in response to M-CSF, as shown by representative microphotograph of migrated cells and by quantification (Figure 2A). By contrast, *Erk2^−/−^* preosteoclasts did not demonstrate alterations in M-CSF-induced migratory capacity.

The initiation of osteoclastic bone resorption depends on osteoclasts' ability to bind to the bone surface through the interaction of cell surface receptors, such as the αvβ3 integrin, with extracellular bone proteins [27], [30], [31], [32]. We examined whether deficiency of *Erk1* or *Erk2* affects αvβ3- and M-CSF-mediated preosteoclast adhesion. We found that fewer *Erk1^−/−^* preosteoclasts adhered to vitronectin during stimulation with M-CSF (Figure 2B), while the expression of integrin αv (CD51) was similar between the different genotypes (data not shown). We found no difference between WT and *Erk2^−/−^* osteoclasts in their capacity to adhere to vitronectin in cell culture.

To functionally assess the consequence of *Erk1* or *Erk2* disruption on osteoclast bone lytic activity, we cultured osteoclasts on dentine slices and examined the number and area of “pits” formed by osteoclast resorptive activity, as previously described [27], [29]. Representative microphotographs of bone resorption are shown (Figure 2C). The quantitative data represents the total resorptive area (Figure 2C, upper panel). The resorptive area per osteoclast is shown quantitatively (Figure 2D). *Erk1^−/−^* osteoclast culture demonstrated a three-fold reduction in resorbed area and the *Erk2^−/−^* osteoclast culture demonstrated an approximate two-fold reduction, as compared to the WT osteoclast culture.

Collectively, these data indicate that *Erk1* positively regulates osteoclast differentiation and M-CSF-mediated migration and pit formation. Deletion of *Erk2* also reduces osteoclast nucleation and bone resorptive activity (albeit less substantially than Erk1). *Erk1^−/−^* cultures show stronger phenotypic changes although Erk2 exists. This may be explained by ERK2 expression in osteoclasts is not high enough to compensate for the loss of ERK1. Nevertheless, our cell culture data suggests important and preponderant contributions of Erk1 toward osteoclast differentiation and function.

### Genetic deletion of *Erk1* in osteoclasts reduces total kinase activity

**Figure 3:**
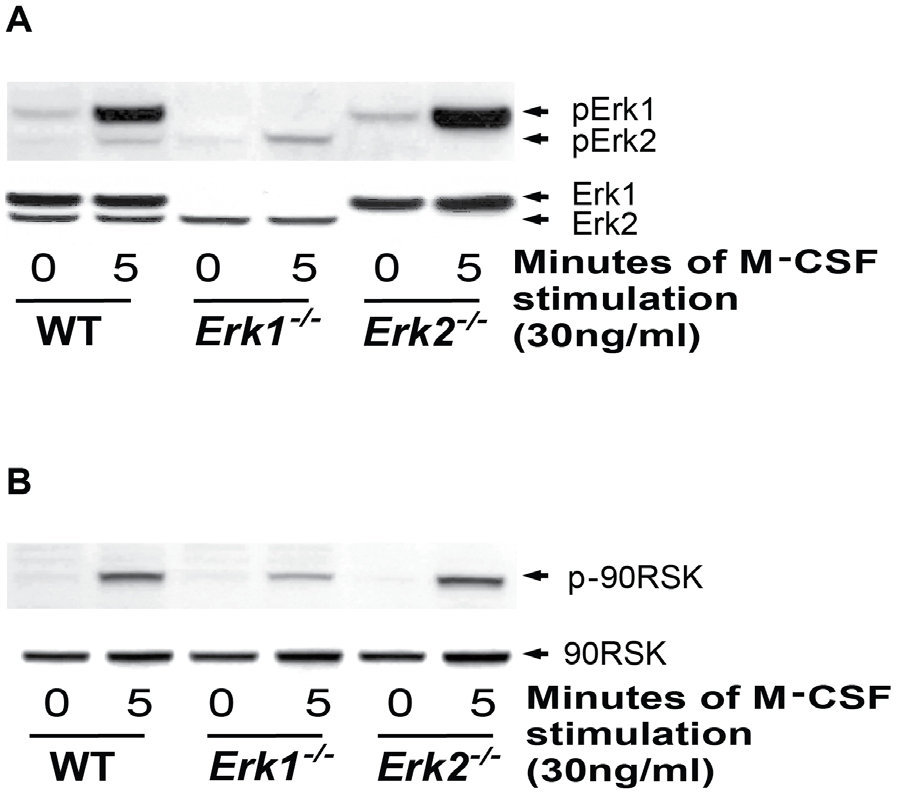
Effect of *Erk1* genetic deletion on MAPK activation in preosteoclasts. Phosphorylation of Erk1/2 (Figure 3A) and p90RSK (Figure 3B) in preosteoclasts of WT, *Erk1^−/−^* and *Erk2^−/−^* mice was measured at the indicated times following stimulation with M-SCF. Representative blots are shown.

To examine biochemical alterations in the MAPK pathway in the context of *Erk1* or *Erk2* disruption, we stimulated cultured preosteoclasts with M-CSF (30 ng/mL), lysed the cells, and extracted protein for western blot. We assessed total and phosphorylated levels of Erk1, Erk2, and p90^rsk^, a downstream Erk1/2 effector. Following M-CSF stimulation, Erk1 phosphorylation increased dramatically in WT and Erk2^−/−^ cells, while no pErk1 was detected in *Erk1^−/−^* cells (Figure 3A). Interestingly, a slight, perhaps compensatory, increase in pErk1 was observed in *Erk2^−/−^* cells, as compared to WT. Similarly, we observed a moderate increase in pErk2 levels in M-CSF-stimulated *Erk1^−/−^* preosteoclasts, as compared to WT cells. As expected, no pErk2 was observed in Erk2^−/−^ cells.

We also found a substantial reduction in phospho-p90^rsk^ in M-CSF-stimulated *Erk1^−/−^* preosteoclasts, as compared to stimulated WT and *Erk2^−/−^* cells (Figure 3B). Since PI3-K has been shown to regulate osteoclast development [33], we also examined phosphorylation of Akt in the cultured preosteoclasts. Similar levels of Akt phosphorylation were observed among WT, *Erk1^−/−^*, and *Erk2^−/−^* preosteoclasts (data not shown), suggesting that the functional aberrancies observed in *Erk1*-deficient osteoclasts are Akt independent. Quantitative analysis of the western blot result is shown in Figure S4.

### Genetic deletion of *Erk1* reduces osteoclast progenitors

**Figure 4:**
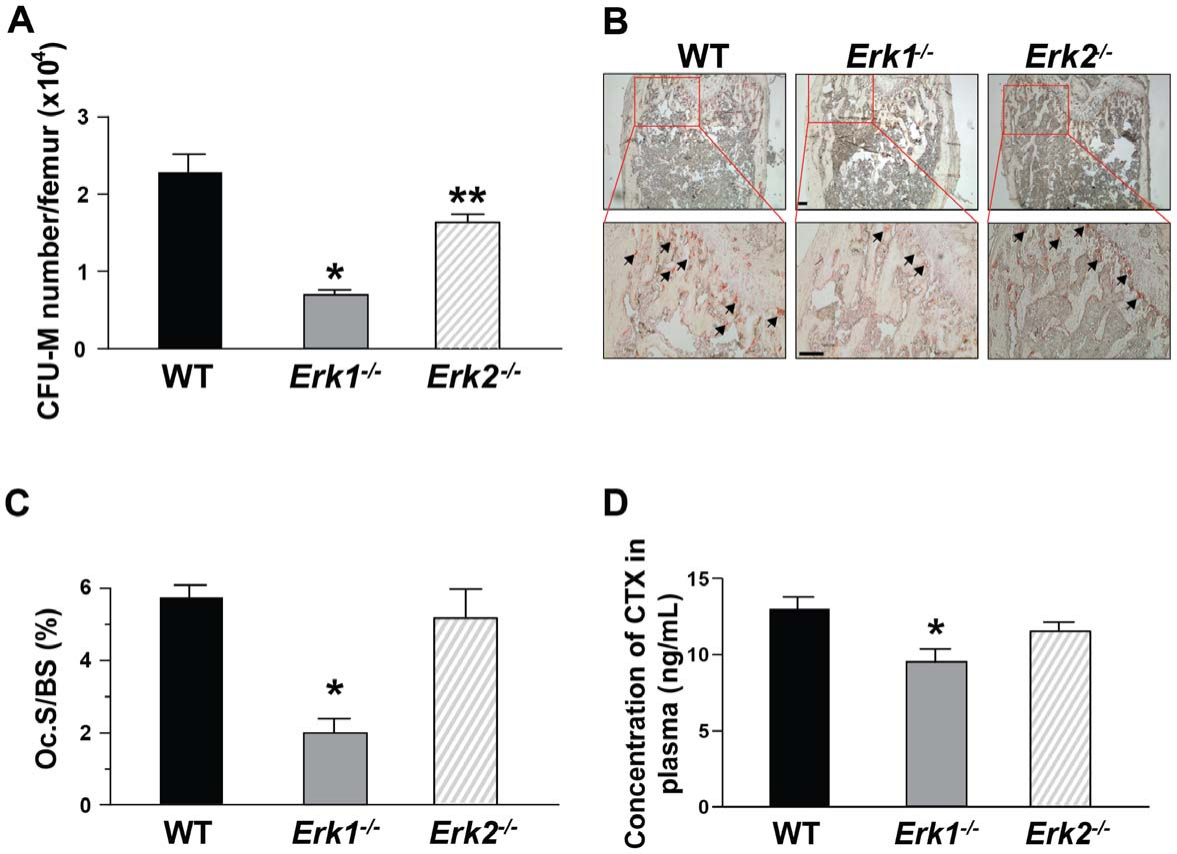
Genetic deletion of Erk1 affects osteoclast progenitor frequency and osteoclast formation *in vivo*. (**A**) BMMNCs of the indicated genotypes were cultured in agar-based media containing M-CSF (30 ng/mL) and RANKL (20 ng/mL) for 7 days and CFU-macrophage (M) were counted based on morphology. Y-axis indicated CFU-M number per femur. Data represents mean ± SEM of triplicate cultures. *P<0.01 for *Erk1^−/−^* vs. WT, **P<0.05 for *Erk2^−/−^* vs. WT and *Erk1^−/−^*, as evaluated by ANOVA followed by post-hoc *t*-tests. Experiments were conducted on three independent occasions with similar results. (**B**) Representative microphotographs (40×, 100× magnification) of WT, *Erk1^−/−^* and *Erk2^−/−^* distal femoral metaphyses following TRACP staining. Arrows indicate selected osteoclasts. Scale bar = 100 µm. (**C**) Data represent the mean ± SEM of 5 independent experiments. Ten high-power fields per experimental mouse were scored. *P<0.01 for *Erk1^−/−^* vs. WT and *Erk2^−/−^*, as analyzed by ANOVA followed by post-hoc *t*-tests. (D) Detection of CTX in plasma of WT, *Erk1^−/−^* and *Erk2^−/−^* mice (N = 4–6 mice in each group).*P<0.05 for *Erk1^−/−^* vs. WT, as analyzed by ANOVA followed by post-hoc *t*-tests.

Since osteoclasts are tissue-specific progeny of the monocyte/macrophage lineage, we quantitatively evaluated the number of macrophage and osteoclast progenitors per femur in *Erk1^−/−^*, *Erk2^−/−^*, and WT mice using established clonogenic assays [27]. Following culture in semisolid media supplemented with osteoclast-promoting cytokines (i.e. M-CSF, RANKL), the number of osteoclast and macrophage progenitor colonies were scored. *Erk1^−/−^* BMMNCs developed approximately one-fourth to one-third fewer macrophage colonies as compared to the number of colonies formed from *Erk2^−/−^* and WT BMMNCs (Figure 4A). Although the osteoclast progenitors are decreased in *Erk2^−/−^* also, the result showed that Erk1 plays a more vital role than Erk2 in osteoclastogenesis. Of note, there was no significant difference in total bone marrow cellularity between *Erk1^−/−^*, *Erk2^−/−^*, and WT mice (data not shown), indicating a primary reduction in the frequency of monocyte/osteoclast progenitor cells in *Erk1^−/−^* bone marrow.

### Reduced TRACP^+^ osteoclast development in *Erk1^−/−^* mice

Given the impaired osteoclast differentiation *in vitro* and reduced number of osteoclast progenitors in *Erk1^−/−^* bone marrow, we next assayed the number of mature osteoclasts in *Erk1^−/−^* mice *in vivo*. The femurs of 8-week old syngeneic WT, *Erk1^−/−^* and *Erk2^−/−^* mice were fixed and embedded in methyl methacrylate, and histological sections from the distal metaphysis were stained with the osteoclast enzyme TRACP. Compared to WT mice, the TRACP^+^ area per low power field (100× magnification) of trabecular surface in *Erk1^−/−^* mice was decreased as qualitatively and quantitatively shown in Figure 4B and Figure 4C, respectively. To determine if deletion of *Erk1* additionally alters osteoblast formation, McNeal staining was performed on the methyl methacrylate processed histological sections, revealing no significant change in osteoblast numbers between WT, *Erk1^−/−^*, and *Erk2^−/−^* bone sections (Figure S3).

### Reduced C-terminal telopeptide of type I collagen (CTX) in *Erk1^−/−^* mice

Type I Collagen, which constitutes more than 90% of the organic matrix of bone, can be degraded and released during osteoclastic bone resorption. Thus, the level of CTX production in plasma is a sensitive marker of bone loss [34]. The level of CTX production in plasma was substantially reduced in *Erk1^−/−^* mice as compared with that in WT and *Erk2^−/−^* mice (Figure 4D), suggesting a reduced osteoclast bone resorptive activity in *Erk1^−/−^* mice *in vivo*.

### Increased bone mineral density in *Erk1^−/−^* mice and *Erk1^−/−^* bone marrow mononuclear cell transplanted recipients

**Figure 5:**
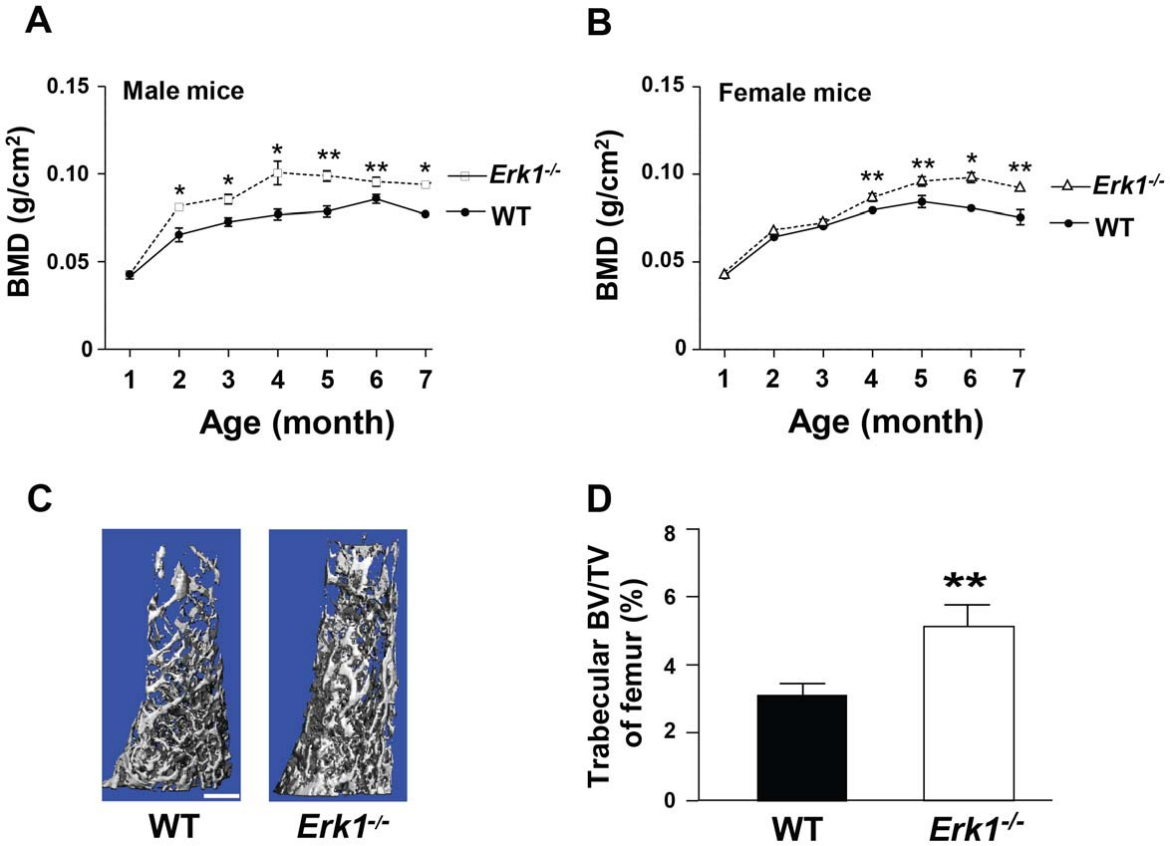
*Erk1^−/−^* mice have increased BMD and BV/TV. BMD of age and sex matched *Erk1^−/−^* and WT mice was measured from birth to 7 months of age. The BMD of male (**A**) and female (**B**) WT and *Erk1^−/−^* mice is shown (N = 5 mice in each group). (**C**) Representative μCT reconstructions of WT and *Erk1^−/−^* femurs are shown. Scale bar = 1 mm. (**D**) Quantitative data comparing the left femur BV/TV between WT and *Erk1^−/−^* mice.

Given that alteration in osteoclast bone resorptive activity affects bone structure, we hypothesized that *Erk1^−/−^* mice would demonstrate increased bone mineral density (BMD). To test this hypothesis, we acquired BMD data using dual-energy X-ray absorptiometry (DEXA) with a Lunar Piximus densitometer. We specifically analyzed a region of interest with a high content of trabecular bone, located proximal to the distal growth plate of the left femur of each mouse. *Erk1^−/−^* male mice demonstrated increased BMD, as measured monthly after birth (Figure 5A). *Erk1^−/−^* female mice also displayed increased BMD as compared to WT littermates (Figure 5B), though the difference was milder than in the male mice. However, the cause of the different BMD between male and female mice remains unclear.

To compare the long bone mass between the WT and *Erk1^−/−^* mice, micro-computed tomography (μCT) was utilized to examine the bone volume and architecture. *Erk1^−/−^* mice exhibited an ∼80% increase (**P<0.05) in trabecular bone volume fraction (BV/TV), (Figure 5C and 5D); an ∼15% increase (**P<0.05) in trabecular thickness, an ∼50% increase (**P<0.05) in trabecular number, and an ∼30% decrease (**P<0.05) in trabecular separation, as compared to the WT controls (Figure S6A–C). This increased bone volume in *Erk1^−/−^* mice is consistent with the increase of BMD in *Erk1^−/−^* mice.

**Figure 6:**
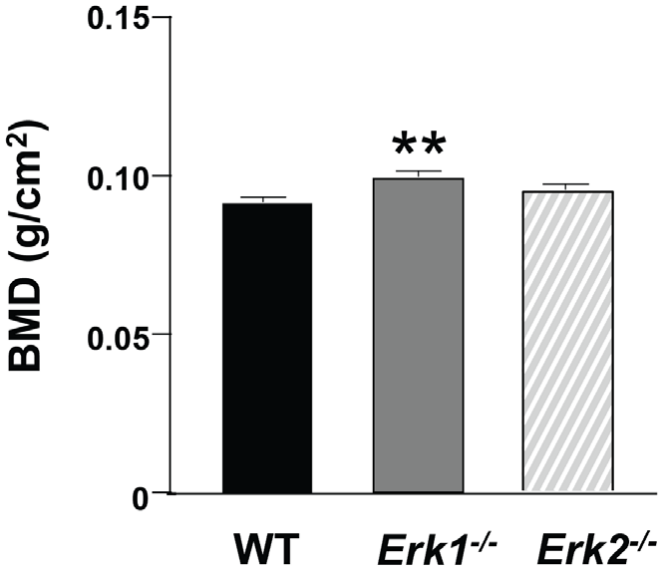
Transplantation of *Erk1^−/−^* bone marrow increases BMD in WT recipients. Six months after transplantation with WT, *Erk1^−/−^* or *Erk2^−/−^*BMMNCs into WT recipient mice, BMD was measured (N = 5 mice in each group). **P<0.05 for *Erk1^−/−^* vs. WT recipients, as analyzed by ANOVA followed with post-hoc *t*-tests.

Based on our tissue culture and *in vivo* findings, we hypothesized that aberrant formation and function of *Erk1^−/−^* osteoclasts directly results in increased BMD. To test this hypothesis, we performed long-term transplantation [26] of BMMNCs from WT, *Erk1^−/−^* and *Erk2^−/−^* mice into lethally-irradiated WT BoyJ recipient mice, thus isolating our analysis to cells derived from the donor hematopoietic cells. For complete reconstitution of hematopoietic cells, we waited six months after the transplantation to confirm successful reconstitution of hematopoietic stem cells and *Erk2* allele deletion by flow cytometry-based detection of CD45.2 expression (data not shown) and by western blot of peripheral blood (Figure S5), respectively. We then measured BMD in the recipient mice, finding that *Erk1^−/−^* bone marrow reconstitution produced increased BMD as compared to WT recipients (Figure 6), while transplantation of *Erk2^−/−^* BMMNCs did not alter BMD as compared to WT recipients. These data imply a marrow-autonomous role for *Erk1* in regulating osteoclast development and bone resorptive activity *in vivo*.

## Discussion

Alterations of the Ras/MAPK pathway have been observed in disease models of Paget's bone disease, bone metastasis, and Neurofibromatosis type 1 (NF1) [35], [36], [37], [38]. Moreover, congenital disorders including Noonan syndrome, Noonan-like/multiple giant cell lesion syndrome, NF1, LEOPARD syndrome, Costello syndrome, and Cardio-facio-cutaneous syndrome carry germline mutations that variously affect genes within the MAPK signaling cascades [39]. These studies broadly implicate deregulation of Ras signals in skeletal pathologies. However, the interaction between and contribution of individual cell lineages (e.g. osteoblasts, osteoclasts, mesenchymal stromal cells) to specific pathologies, as well as their potential dependence on a specific Erk isoform, require further delineation.

Previously, we found that the Mek-Erk inhibitor PD98059 reduces osteoclast development and belt formation [29]. Since PD98059 inhibits phosphorylation of both Erk1 and Erk2, and perhaps exhibits a high degree of non-selectivity at its applied concentration, we were unable to delineate if Erk1, Erk2, both, or, potentially, neither isoform modulates osteoclast differentiation. Here, we show that genetic disruption of *Erk1* versus *Erk2* preferentially impairs osteoclast formation and function *in vitro* and *in vivo*. Important to note, this phenotype arises in a marrow-autonomous manner, implicating *Erk1's* importance to the marrow-derived osteoclast and its regulation of normal bone physiology. Although we show that Erk2 cannot compensate, either functionally or biochemically, for loss of *Erk1* in the osteoclast, we still suspect that Erk2 can positively contribute to osteoclastogenesis and osteoclast function. Supporting this notion, we have found that *Erk1/2* dual disruption ablates early myelopoiesis and precludes the study of osteoclastogenesis, a phenotype which will be reported in detail elsewhere. By contrast, Erk1 appears largely capable of compensating for *Erk2* disruption in the observed biochemical and functional phenotypes. Thus, we have concluded that Erk1 plays the preponderant role in regulating osteoclastogenesis and osteoclast function. However, it remains unknown whether this phenotype results from isoform functional specificity or solely from osteoclast-intrinsic dependence on increased Erk1 expression and/or activation.

Biochemical, pharmacological, and genetic models have broadly implicated ERK1/2 activity in proliferation, survival, migration, and protein synthesis in diverse cell types (reviewed in [40]). However, lineage- and isoform-specific functions are less known, and genetic murine studies have been limited, thus far demonstrating *Erk1-*specific requirements for thymocyte development [18], erythropoiesis [41], adipogenesis [19], and skin tumor development [17]. Other studies have suggested a negative regulatory role for Erk1, whereby *Erk1* disruption enhances fibroblast and neuron function by dis-inhibiting Mek-Erk2 signals [42], [43]. These data suggest the preponderance of Erk2 over Erk1 function in these cell types. Accordingly, *Erk2* disruption is embryonically lethal [44] while, grossly, *Erk1* ^−/−^ mice demonstrate no profound phenotypes [18]. However, as reported elsewhere and as we show here for the osteoclast, homeostatic processes in particular organ systems may depend upon Erk1, and generalizations as to Erk1 versus Erk2's functional importance should not be derived from observations in one cell type. Our results showed that Ekr1 had higher expression level than Erk2 in preosteoclasts; it is most likely that Erk1 has higher affinity than Erk2 does for the antibody.

Different cell lineage- and isoform-specific studies *in vitro* and *in vivo* will produce crucial insights into the subtleties of MAPK signaling, thus informing therapeutic strategies. Accordingly, ERK isoform-specific chemical inhibition may prove pivotal to targeted therapy. Though bisphosphonates have been the cornerstone of osteoporosis therapy since the 1960's, recent work has focused on more selective compounds [45]. Molecular targets to Ras itself, such as farnesyl transferase inhibitors, have been disappointing, as K-ras and N-ras, the isoforms prevalent in myeloid lineages, do not depend on farnesylation. Because disruption of *Erk1* dampens osteoclast function to a degree sufficient to increase bone mineral density *in vivo*, an Erk1-targeted chemical kinase inhibitor may be an effective therapeutic agent for the diminished bone mineral density found in multiple skeletal pathologies, including hormonal loss-dependent osteoporosis. This strategy may selectively modulate osteoclast function while producing fewer off-target and detrimental effects potentially associated with dual Erk1/2 inhibition. Of note, a recent crystal structure of human ERK1 revealed substantial differences in D-motif and backside binding sites, as compared to ERK2, indicating the feasibility of a selective ERK1 inhibitory agent [46].

In conclusion, osteoclastogenesis and osteoclast functions depend upon Erk1 to a greater degree than Erk2, and singular genetic inhibition of *Erk1* mitigates osteoclast function *in vitro* and *in vivo*. This loss-of-function phenotype appears to proceed in a marrow autonomous manner, thus implicating a primary defect within the *Erk1*-disrupted osteoclast. These results uniquely demonstrate a positive regulator role for Erk1 while suggesting the potential for Erk isoform-targeted therapy of osteoporotic conditions.

## Supporting Information

Figure S1 Genotypic analysis. PCR was performed to confirm the genotype of WT (lane 1), *Erk1^−/−^* (lane 2) and *Mx1Cre^+^ Erk2^fl/fl^* (lane 3) mice.
(TIF)

Figure S2 Western Blot. Representative western blot of three independent experiments shows the total protein level of Erk1 or Erk2 in bone marrow mononuclear cells (BMMNCs) and osteoclast. β-actin was utilized as a loading control.
(TIF)

Figure S3 (**A**) Representative microphotographs (100× magnification) of WT, *Erk1^−/−^* and *Erk2^−/−^* distal femoral metaphyses following McNeal Staining. Arrows indicate selected osteoblasts. (**B**) Data represent the mean ± SEM of 5 independent experiments. Six high-power fields per experimental mouse were scored. Scale bar = 50 µm.
(TIF)

Figure S4 Quantitative evaluation of Erk1 phosphorylation over ß-actin level of Figure 3. (**A**), Erk2 phosphorylation (**B**), and phosphorylation of p90RSK (**C**) in WT, *Erk1^−/−^* and *Erk2^−/−^* preosteoclasts is shown.
(TIF)

Figure S5 Western blot shows the protein levels of Erk1 and Erk2 in peripheral blood of the WT and *Erk1^−/−^* transplanted WT recipients. Scale bar =  1 mm.
(TIF)

Figure S6 Quantitative data comparing the left femur Tb.Th, Tb.N and Tb.Sep between WT and *Erk1^−/−^* mice (N = 5 in each group). **P<0.05 for *Erk1^−/−^* vs. WT mice.
(TIF)
