# Supplementary figures and images for: Erk1 Positively Regulates Osteoclast Differentiation and Bone Resorptive Activity
Source: PLoS One. 2011 Sep 22;6(9):e24780. doi: 10.1371/journal.pone.0024780 (PMC3178550; doi:10.1371/journal.pone.0024780)

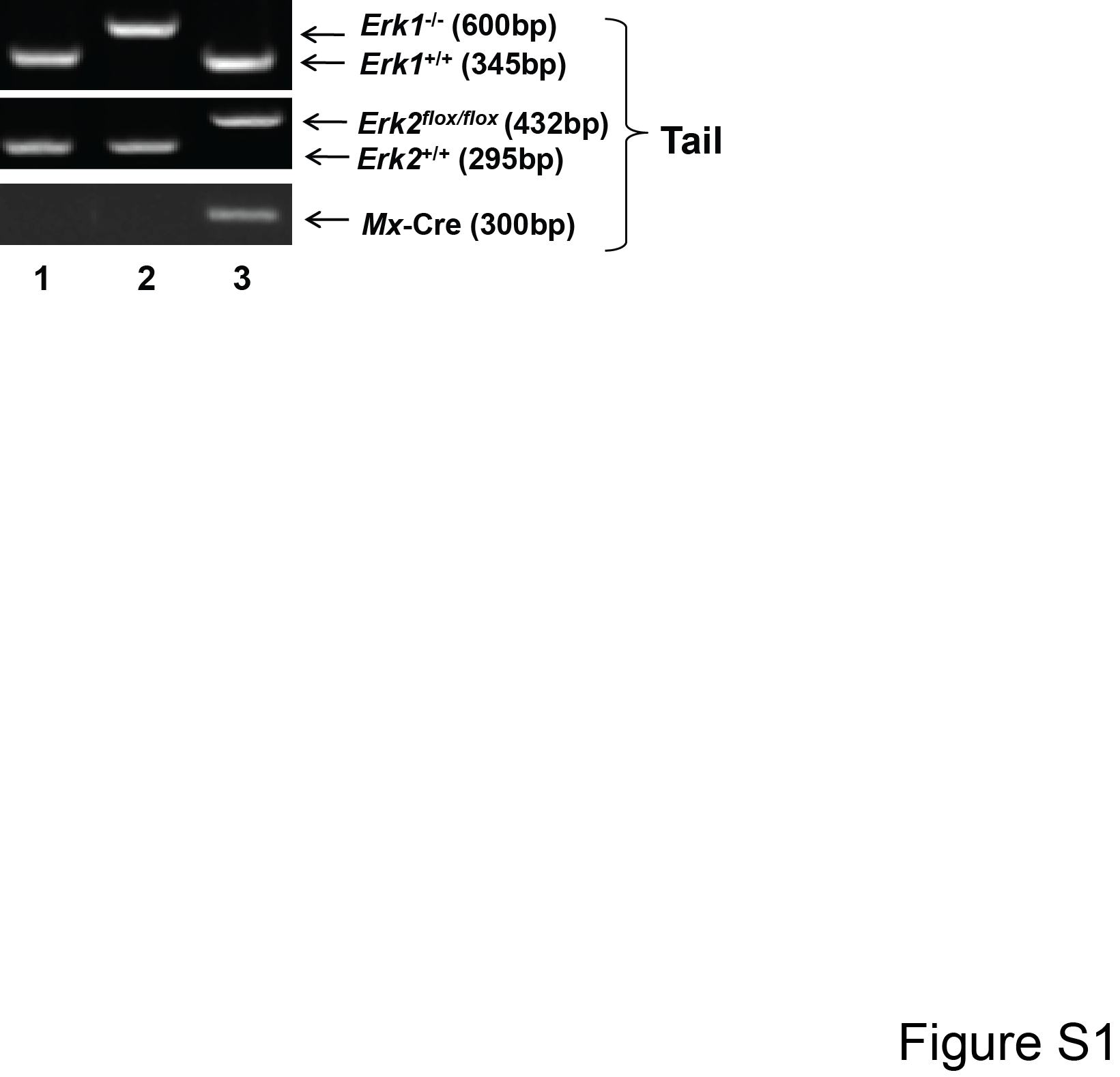

Supplement: Figure S1 — Genotypic analysis. PCR was performed to confirm the genotype of WT (lane 1), Erk1−/− (lane 2) and Mx1Cre+ Erk2fl/fl (lane 3) mice. (TIF) [file pone.0024780.s001.tif]

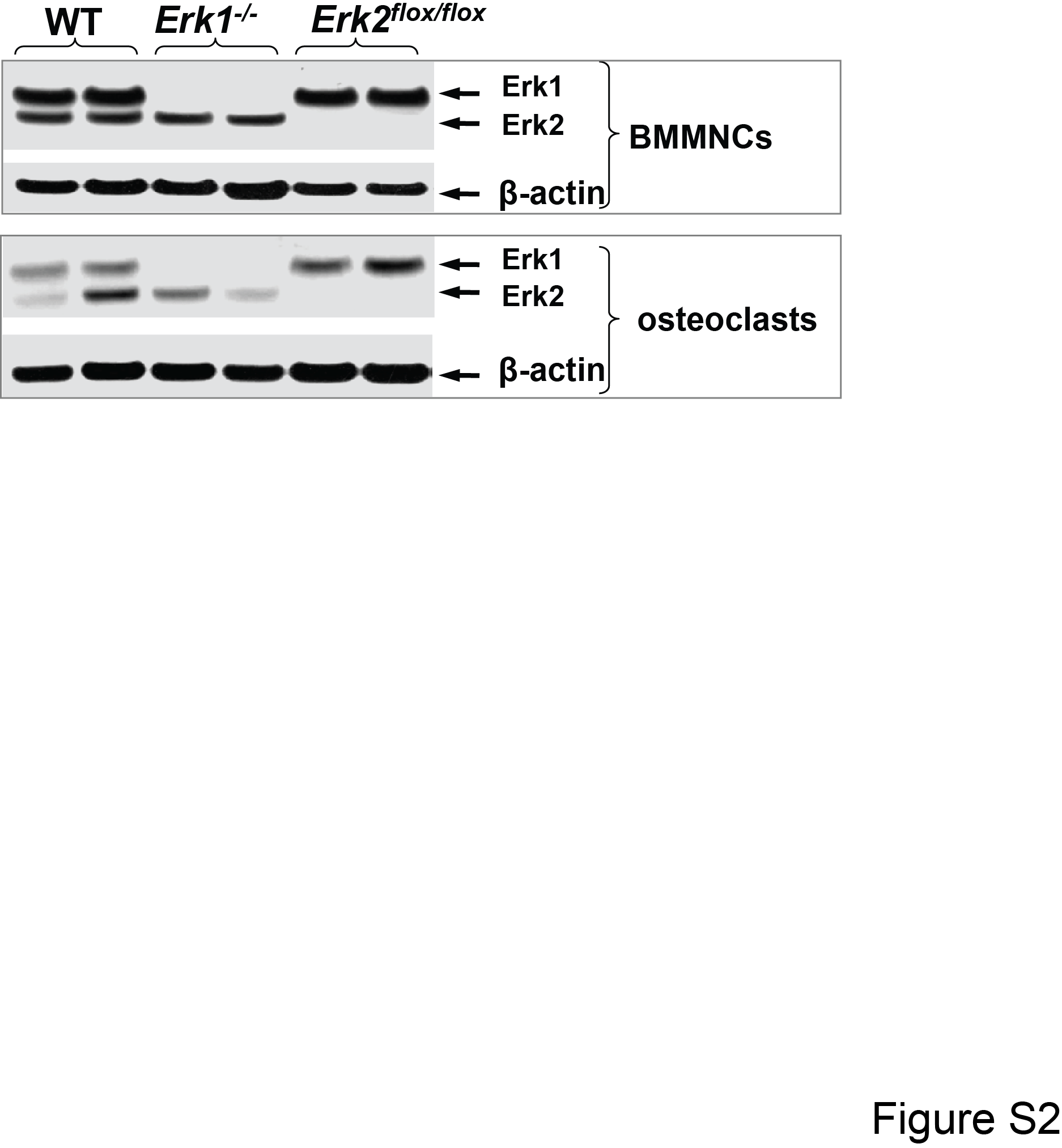

Supplement: Figure S2 — Western Blot. Representative western blot of three independent experiments shows the total protein level of Erk1 or Erk2 in bone marrow mononuclear cells (BMMNCs) and osteoclast. β-actin was utilized as a loading control. (TIF) [file pone.0024780.s002.tif]

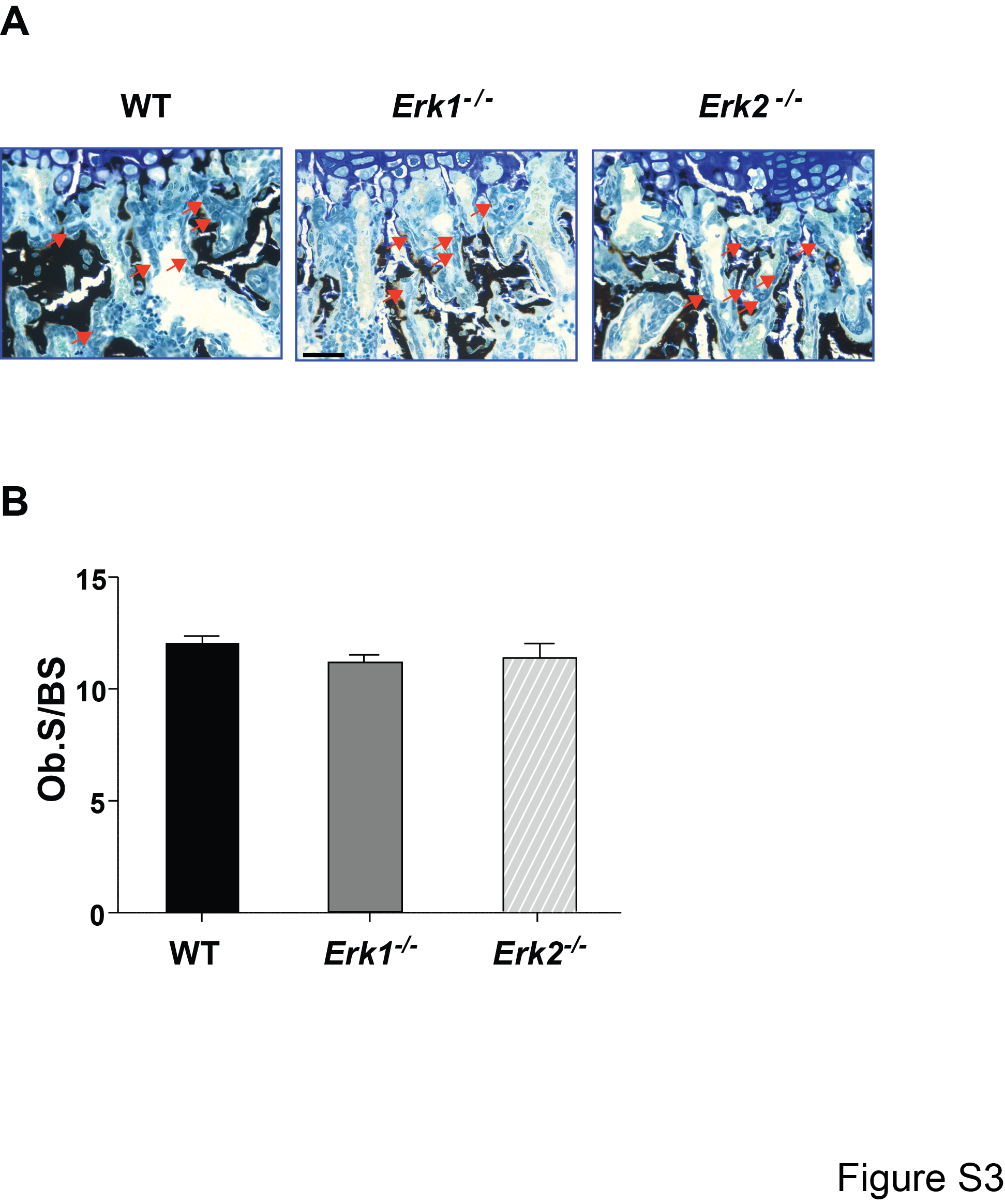

Supplement: Figure S3 — (A) Representative microphotographs (100× magnification) of WT, Erk1−/− and Erk2−/− distal femoral metaphyses following McNeal Staining. Arrows indicate selected osteoblasts. (B) Data represent the mean ± SEM of 5 independent experiments. Six high-power fields per experimental mouse were scored. Scale bar = 50 µm. (TIF) [file pone.0024780.s003.tif]

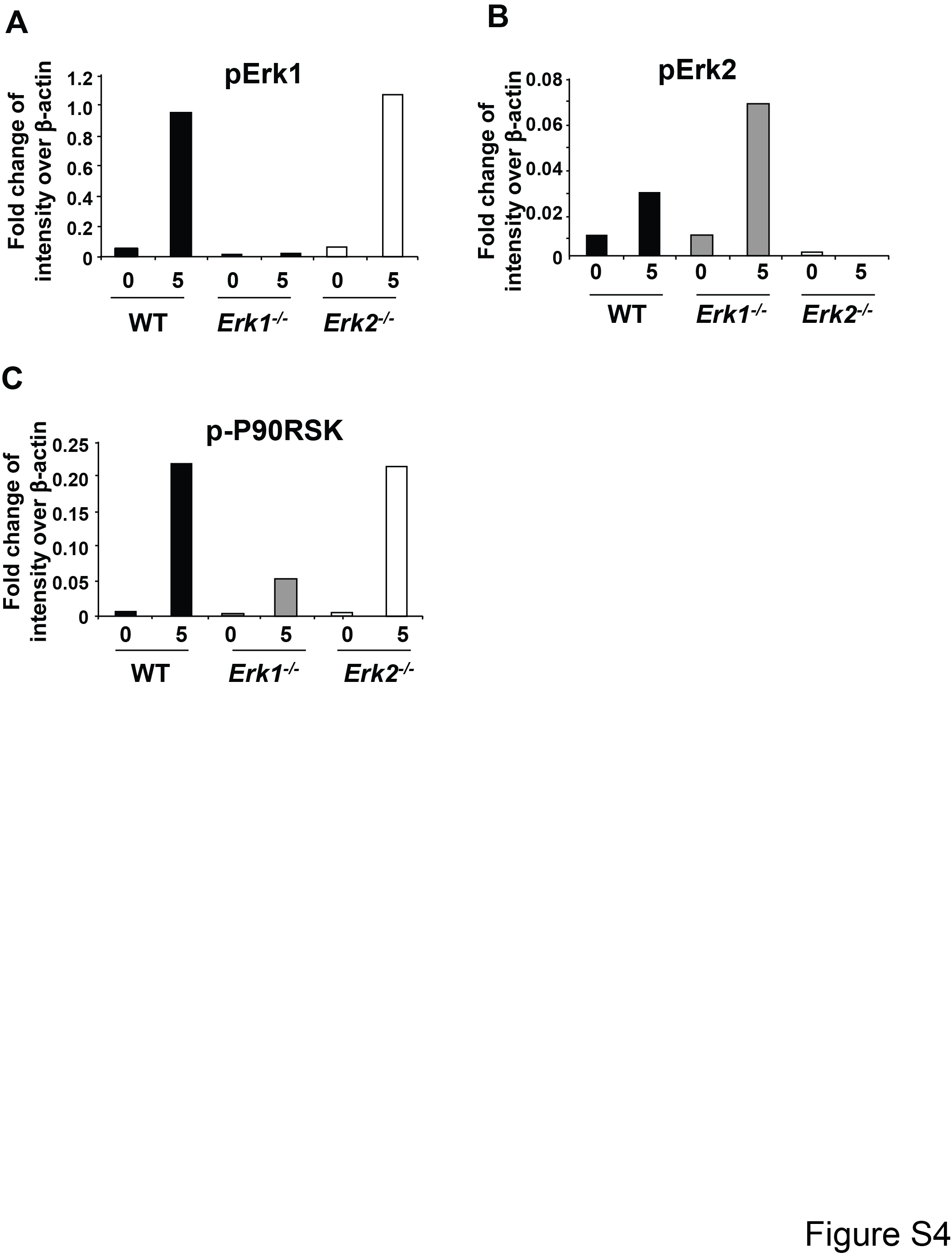

Supplement: Figure S4 — Quantitative evaluation of Erk1 phosphorylation over ß-actin level of Figure 3. (A), Erk2 phosphorylation (B), and phosphorylation of p90RSK (C) in WT, Erk1−/− and Erk2−/− preosteoclasts is shown. (TIF) [file pone.0024780.s004.tif]

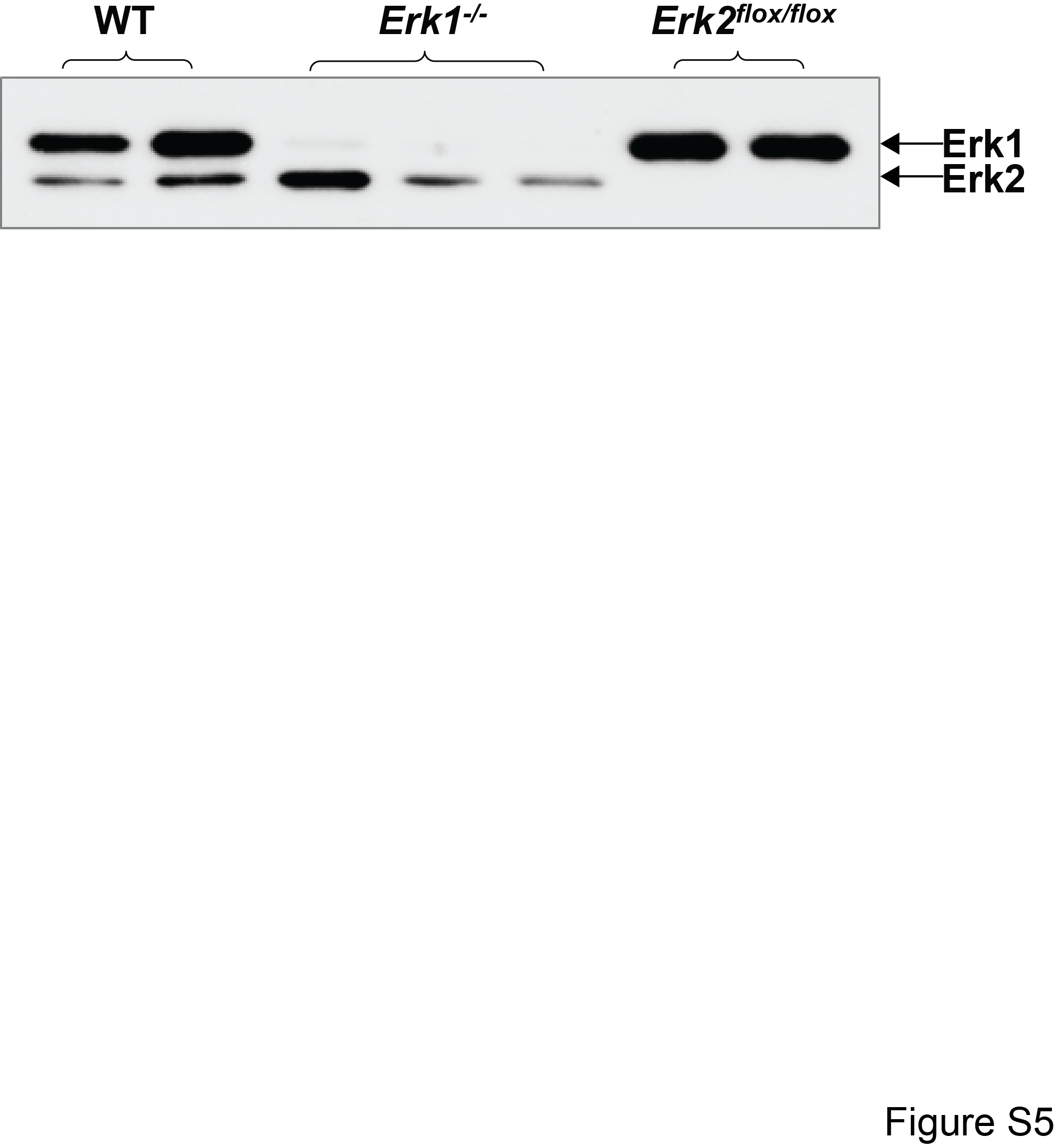

Supplement: Figure S5 — Western blot shows the protein levels of Erk1 and Erk2 in peripheral blood of the WT and Erk1−/− transplanted WT recipients. Scale bar = 1 mm. (TIF) [file pone.0024780.s005.tif]

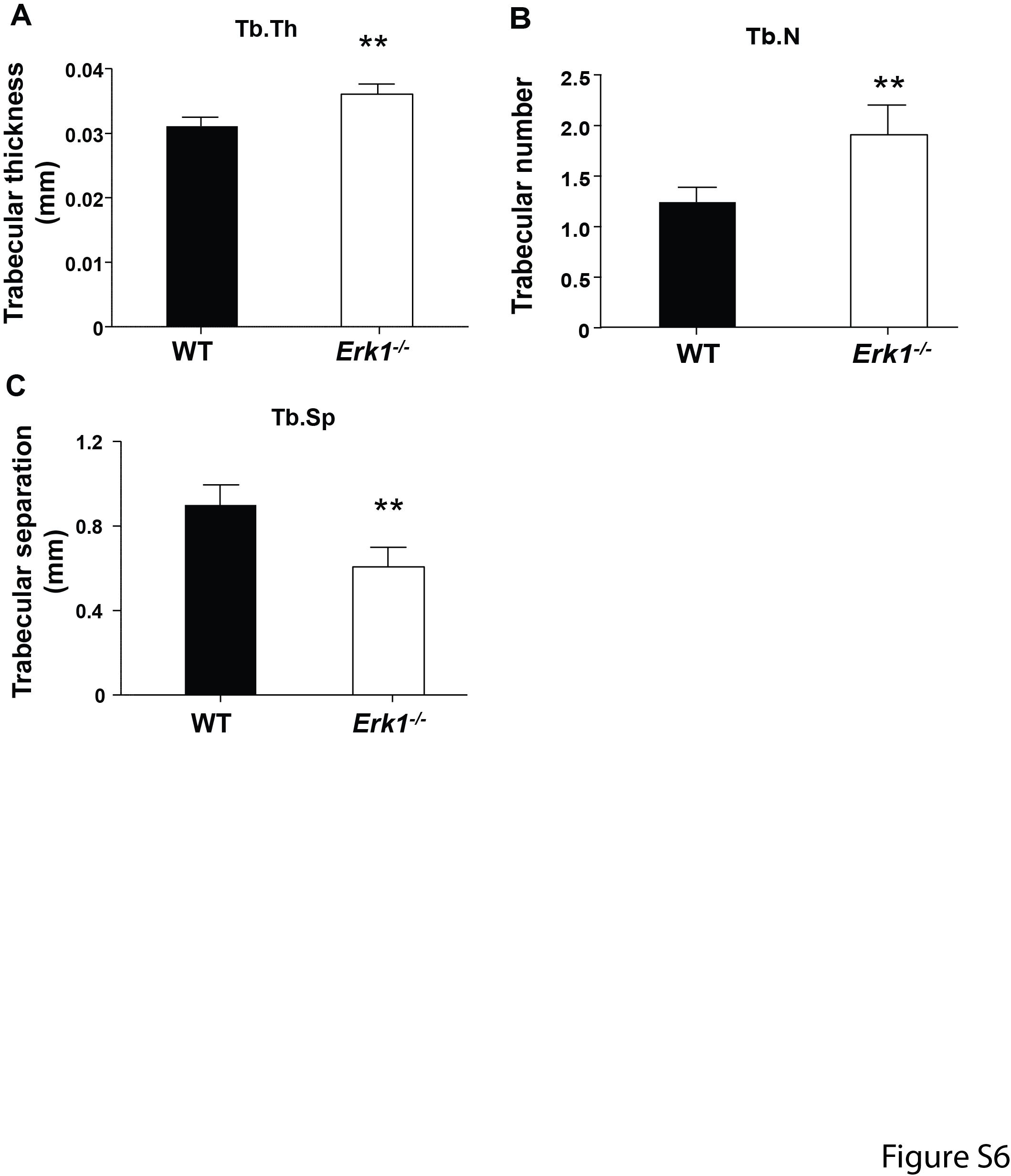

Supplement: Figure S6 — Quantitative data comparing the left femur Tb.Th, Tb.N and Tb.Sep between WT and Erk1−/− mice (N = 5 in each group). **P<0.05 for Erk1−/− vs. WT mice. (TIF) [file pone.0024780.s006.tif]
